# Cooperation program for volunteer medical students for training in pediatric cardiopulmonary resuscitation and accident prevention in Honduras

**DOI:** 10.1186/s13104-020-04962-1

**Published:** 2020-02-27

**Authors:** Helena Garreta, César Gutiérrez, Paula Greciano, Claudia Riber, Javier Urbano, Jesús López-Herce

**Affiliations:** 1 Departamento de Salud Pública y Materno-Infantil, Facultad de Medicina, Universidad Complutense de Madrid, Madrid, Spain; 2 Servicio de Cuidados Intensivos Pediátricos, Hospital General Universitario Gregorio Marañón Madrid, Madrid, Spain; 3 Red de Salud Maternoinfantil y del Desarrollo, Instituto Carlos III, Madrid, Spain

**Keywords:** Cooperation, Health volunteer, Cardiopulmonary resuscitation, Children medical education

## Abstract

**Objectives:**

To analyze a training program in accident prevention and care and Pediatric Basic Cardiopulmonary Resuscitation taught by medical students.

**Results:**

Medical students were trained as instructors. Four courses of were launched in Honduras, and the results were analyzed through a theoretical and practical evaluation and an anonymous survey. The volunteer experience for the students and the benefits to the population were positively valued. 37 students received the training. The score in the initial theoretical evaluation was 5.9 of 17 and in the final 10.5, p < 0.001. 89.1% and 91.9% of the students achieved adequate practical learning in basic Cardiopulmonary Resuscitation for children and infants respectively. The course was rated excellently by the students. We conclude that a training program in accident prevention and care and Pediatric Basic Cardiopulmonary Resuscitation taught by medical students could be useful in a cooperation health program.

## Introduction

Honduras is the second country with the lowest Human Development Index (HDI) in Latin America. Worldwide, it is ranked 130 out of 188 countries [1]. The country presents very high levels of economic inequality. Access to health systems remains deficient for the majority of the Honduran population: it is estimated that 18% do not have access to healthcare and almost 9 out of 10 people are not covered by any type of insurance [2].

It is important to orient students’ attitudes toward the poorest populations and promote an ethic of social responsibility. Therefore, incorporating volunteering into medical degree programs offers the opportunity to create awareness and interest to help those most in need [3].

Volunteering in Honduras has achieved improvements in very diverse areas [4]. Due to the high infant mortality rate (17.4% in children under 1 year of age and 20.4% in children under 5 years of age) [5], the pediatric population is one of the main focuses of volunteer care. The main causes of infant death are infections, malnutrition, anemia, malformations and accidents [6]. Cardiac arrest in countries with a medium and low HDI has a worse prognosis than in countries with a high HDI, and these results are partly due to poor training in prevention measures and in cardiopulmonary resuscitation (CPR) [7]. To improve the results of CPR, the dissemination of international recommendations and the training of health professionals and the general population is essential [8].

There are previous experiences of CPR training in Latin America with very positive results [9]. Although to achieve widespread CPR training it is necessary to dedicate multiple teaching centers and do a chain training, teaching basic CPR to the general population can allow immediate assistance in the first minutes of cardiac arrest and improve the prognosis [10].

The objectives of this study were firstly to analyze the ability of medical students to act as health trainers in a health volunteer project, to evaluate the usefulness of accident prevention and CPR courses given by medical students to the Honduran population and evaluate the experience of health volunteering by medical students.

## Main text

The project was carried out in several stages.

### 1st: Design of the volunteer project and the course on prevention and care of accidents and basic cardiopulmonary resuscitation for the general population

A volunteer project was designed in Honduras to be carried out by the Medicine students of the Complutense University of Madrid with the non-profit association “Collaboration and Effort Association” (ACOES) that included training activities in basic pediatric cardiopulmonary resuscitation and prevention and accident care directed to the general population and taught by previously trained students.

A 10-h course model of prevention and care of accidents and pediatric basic cardiopulmonary resuscitation was designed for the general population adapted to Honduras (Additional file 1).

The material to teach the courses (two mannequins of pediatric cardiopulmonary resuscitation of children (ResusciJuniorR, Laerdal) and infant (ResuscibabyR, Laerdal), and a basic kit) was obtained thanks to an aid for the health cooperation of the Health Research Institute of the Hospital Gregorio Marañón of Madrid.

A maximum number of 15 students per course was established to allow sufficient practice and correction of resuscitation techniques in each of the students.

A theoretical evaluation was designed to apply before and after the course, adapted from the evaluation of the basic CPR course of the Spanish Pediatric and Neonatal CPR Group. The theoretical evaluation consisted of a questionnaire of 17 multiple-choice questions on practical clinical cases with a single correct answer. For the practical evaluation, the sheet designed by the Spanish Pediatric and Neonatal CPR Group was used, which evaluates between 1 (not performed) and 5 (performed perfectly) each of the basic cardiopulmonary resuscitation maneuvers (assessment of consciousness, opening of the airway, ventilation, cardiac massage and global action). A score equal to or greater than 3 was considered adequate in the practical evaluation (corresponding to an effective cardiopulmonary resuscitation that could achieve the recovery of the child without serious sequelae). The anonymous evaluation survey of the course was adapted by the Spanish Pediatric and Neonatal CPR Group students (Additional file 2).

### 2nd: Training of trainers course

The medical students had previously performed a pediatric intermediate cardiopulmonary resuscitation course accredited by the Spanish Pediatric and Neonatal Cardiopulmonary Resuscitation Group.

A course of trainers in accident prevention and pediatric basic cardiopulmonary resuscitation was designed, which was held in June 2017 with the objective that students acquire theoretical and practical knowledge about the characteristics and handling of the material, teaching techniques and methodology of the CPR and prevention courses. The course with a duration of 26 h was held in 4 days (Table 1). In addition to the training as trainers in basic pediatric CPR, as well as in accident prevention and care, a day was dedicated to training in volunteering.

**Table 1:** Course program of trainers in pediatric basic cardiopulmonary resuscitation and accident care

Date	Formation
First day (6.5 h)	Introduction to the volunteer experience • General volunteering. Recommendations and advice • Explanation of the activities of ACOES Madrid
Second day (6.5 h)	Basic CPR instructors course 1. Introduction. Methodology of the course of monitors 2. Program and methodology of the prevention and CPR course 3. Characteristics of teaching CPR to non-health adults 4. Preparation of clinical cases and evaluation of students and the course 5. Theoretical classes 6. Practical classes
Third 3 (6.5 h)	Training course in accident prevention and first aid 1. Content and methodology of the accident prevention course 2. Practice of discussing situations of accident risk and prevention and contents of the kit. Discussion of the most frequent accidents in children in the home, street and school and preventive measures 3. Explanation and practice of the content of the kit 4. Traumatism, convulsions and intoxications care practice 5. Practice of attention to hemorrhages, wounds and burns
Fourth 4 (6.5 h)	Simulation of accident prevention course and CPR • Simulation of a complete course. Evaluation and correction by the tutors

### 3rd: Development of volunteer activities in Honduras

- Four courses were taught, forming a total of 37 individuals (3 courses with 9 students and 1 course with 10 students), 23 women and 14 men, aged between 16 and 40 years. Each course was developed by three or four volunteers. The occupation of the students was: 21 baccalaureate students, 7 nursing students, 5 housewives collaborating with ACOES, 3 firefighters and 1 teacher. All carried out the theoretical and practical evaluations and the evaluation of the course by the students.
- The activities of the training project were combined with other health care activities in collaboration with Honduran doctors collaborating with ACOES (medical examinations, pharmacy organization, informative lectures on health education about family planning, sexually transmitted diseases, nutrition and first care of the baby to 200 mothers and fathers of children).

### 4th: Evaluation of the results of the training program and the volunteer experience

A survey was developed to assess the experience of the volunteers. The survey was prepared by the students themselves because they did not find a similar scale in the bibliographic review carried out.

The qualitative data are expressed in frequencies, and the quantitative ones in means and standard deviations or in medians and interquartile range (RIC) according to the size and distribution of the population. The comparison of the results of the theoretical evaluations was carried out a before-after test with the parametric Student’s T test. A value of p < 0.05 was considered significant.

### Results of accident prevention and CPR course

In the initial theoretical evaluation the score was 5.9 ± 2.6 and in the final 10.5 ± 3, on a maximum of 17 (p < 0.001). 15 students (40.5%) achieved a score equal to or higher than 12 in the final theoretical evaluation.

In the practical evaluation, 89.2% of the students reached an adequate level in basic CPR skills in children and 91.9% in infants. The evaluation of the course by the students (score from 1 to 5) is shown in Table 2.

**Table 2:** Course evaluation by students

Evaluation	Results
Theoretical content	4.8 ± 0.6
Information	91.8% adequate, 2.7% few, 5.4% excessive
Methodology	4.8 ± 0.3
Practical classes	4.8 ± 0.4
Practical time	91.4% adequate, 8.6% excessive
Number of students	80% adequate, 5,7% few 14,3% excessive
Training of instructors	4.9 ± 0.2
Coordination between teachers	4.8 ± 0.3
Course duration	79.4% adequate, 11.7% escasa, 8.8% excessive
Schedule	68.6% adequate, 17.1% escaso, 14.3% excessive

#### Evaluation of the volunteer experience by medical students

The results of the survey on the volunteer experience (score from 1 to 5 points) are shown in Table 3.

**Table 3:** Evaluation of the volunteer experience (median and IQR)

Parameters	Median	IQR
Values acquired during volunteering	5	4.2–5
Organizational capacity	4	4–4.7
Ability to give to others	5	4.2–5
Patience	4	3.2–4.7
Empathy	5	4.2–5
Coexistence capacity	5	5–5
Respect for different people, cultures and religions	5	5–5
Open attitude without prejudice	5	5–5
Sensibility with the most disadvantaged countries	5	4.2–5
Critical view of society	5	5–5
Relativization of problems	5	4.2–5
Activities carried out after volunteering		
Promotion of volunteering in their social environment	5	4.2–5
Continuation as a volunteer in an NGO	2	1.2–4.2
Maintenance of personal relationships created during the stay	2	2–4.2
Benefits that volunteers bring to local people and institutions		
Work capacity, organization and perseverance	4.5	4–5
Application of knowledge and perspectives	5	5–5
Increase motivation and appreciation towards their work	5	4.2–5
Learning about other cultures and other ways of living and thinking	5	5–5
Expectations for a change in society	4	4–4.7

*IQR* interquartile range

### Discussion

This is the first study that analyzes the realization of a training course in accident prevention and CPR to population of a developing country by medical students. Our results show that medical students, if they receive theoretical and practical training and train as instructors, are able to carry out this training work. On the other hand, our study shows the personal and professional benefits that the volunteer activity supposes for the students of Medicine.

It is vital to follow a structured system of training, both for medical students to be instructors and for the Honduran population.

This system of theoretical and practical training, although it requires an important effort, allows to adequately train medical students, who are able to participate adequately in the training and health care programs within a volunteer program. It is important to adapt the program to the characteristics of the population to be studied.

#### Accident prevention and cardiopulmonary resuscitation courses

The majority of students achieved sufficient practical training in basic cardiopulmonary resuscitation skills, demonstrating the usefulness of practical training.

All the students who received the courses evaluated the content and the methodology used very positively. A high proportion of the students affirmed that they felt prepared for the performance of a pediatric CPR.

#### Volunteer experience

The volunteer experience is an unregulated activity that depends mainly on the initiative of some students and teachers. Our experience can serve as a model of volunteer health projects that allow medical students to have an opportunity to participate in a training project within the curriculum of the degree of Medicine, which is compatible with other cooperation activities as occurred in our case.

The results of the questionnaire on volunteering objectify the usefulness of volunteering for medical students in the acquisition of values of coexistence, critical vision of society, respect for different people, cultures and religions, and development of an open attitude and without prejudice.

### Conclusions

We conclude that medical students, after a process of training as instructors, are able to participate adequately in the training and health care programs within a health volunteer program. Accident prevention and pediatric cardiopulmonary resuscitation courses improve the knowledge and practical skills of the Honduran population. The experience of health volunteering is useful at a personal and academic level for medical students. A structured survey can be useful to evaluate the volunteer experience of students participating in a cooperation project.

## Limitations

A fundamental problem of the volunteer programs is the continuity of the activities in the medium and long term. On the other hand, the short duration of the stay of the Spanish volunteers has limited the possibilities of carrying out the supervision task of the trained students. The activity is part of a broader global plan that aims to train local instructors that allow long-term program maintenance.

The survey designed can be useful to evaluate the experience of volunteering of medical students, but it is necessary to evaluate it in a greater number of students.

## Supplementary information

**Additional file 1.** Model of prevention and care courses for accidents and pediatric basic cardiopulmonary resuscitation for the general population.

**Additional file 2.** Anonymous evaluation survey accident prevention in children and pediatric cardiopulmonary resuscitation course.

## Data Availability

Anonymous data of the results of the evaluations could ask for the authors.

## Abbreviations

CPR: Cardiopulmonary resuscitation
ACOES: Collaboration and Effort Association
RIC: Interquartile range
HDI: Human Development Index

## References

[1] United Nations Development Programme. Human Development Reports. 2016. http://hdr.undp.org/sites/default/files/HDR2016_SP_Overview_Web.pdf.

[2] Carmenate Milian L, Herrera Ramos A, Ramos Caceres D, Lagos Ordonez K, Ordonez TL, Valladares CS (2017). Situation of the health system in honduras and the new proposed health model. Arch Med.

[3] Dussán KB, Leidal A, Corriveau N, Montgomery D, Eagle KA, LaHood BJ (2017). Increasing medical trainees’ empathy through volunteerism and mentorship. J Med Educ Curric Dev..

[4] Ropain M. Estudio sobre la situación del voluntariado en honduras: logros, retos y potenciales de un compromiso solidario. 2010 . http://www.undp.org/content/dam/honduras/docs/publicaciones/EstudioSobreSituacionVoluntariadoHonduras.pdf. Access 12 March 2018.

[5] PAHO/WHO Data—Child and maternal mortality. 2018. http://www.paho.org/data/index.php/es/analisis/perfiles-de-salud/275-perfiles-nacional-mortalidad-infantil-materna.html. Access 12 March 2018.

[6] PAHO/WHO Data—Main causes of death. 2018. http://www.paho.org/data/index.php/es/mnu-mortalidad/principales-causas-de-muerte.html. Acceso 11 Marzo 2018.

[7] López-Herce J, Matamoros MM, Moya L, Almonte E, Coronel D, Urbano J, Carrillo Á (2017). Paediatric cardiopulmonary resuscitation training program in Latin-America: the RIBEPCI experience. BMC Med Educ.

[8] López-Herce J, Rodríguez A, Carrillo A (2017). Novedades en las recomendaciones de reanimación cardiopulmonar pediátrica. An Pediatr.

[9] Urbano J, Matamoros MM, López-Herce J, Carrillo AP, Ordóñez F, Moral R (2010). A paediatric cardiopulmonary resuscitation training project in Honduras. Resuscitation..

[10] Carrillo Álvarez Á, López-Herce Cid J, Moral Torrero R, Sancho Pérez L (1999). Enseñanza de la reanimación cardiopulmonar básica pediátrica en la Licenciatura de Medicina y Cirugía. An Esp Pediatr.

