# Supplementary material for: Cooperation program for volunteer medical students for training in pediatric cardiopulmonary resuscitation and accident prevention in Honduras
Source: BMC Res Notes. 2020 Feb 27;13:111. doi: 10.1186/s13104-020-04962-1 (PMC7045480; doi:10.1186/s13104-020-04962-1)
Supplement: Supplementary file 1 — Additional file 1. Model of prevention and care courses for accidents and pediatric basic cardiopulmonary resuscitation for the general population. [file 13104_2020_4962_MOESM1_ESM.docx]

**Additional file 1**

**Model of prevention and care courses for accidents and pediatric basic cardiopulmonary resuscitation for the general population**

| **First day** (4.5 h) | **Second day** (5.5 h) |
| --- | --- |
| - Presentation and introduction of the course. - Theoretical evaluation prior to the course - Theoretical training:   - Prevention of accidents and  cardiorespiratory arrest.  - Material of the first aid kit.   - Theoretical-practical training:   - Initial management of traumatisms,  convulsions and poisonings.  - Initial management of wounds,  nosebleeds, burns, bites and bites. | - Theoretical training:   - Basic cardiopulmonary resuscitation in  the infant and child.   - Practical training and simultaneous practical evaluation:   - Basic CPR in the child  - Basic CPR in the infant   - Theoretical evaluation after completion of the course. - Correction and resolution of doubts. |
