# Supplementary material for: Cooperation program for volunteer medical students for training in pediatric cardiopulmonary resuscitation and accident prevention in Honduras
Source: BMC Res Notes. 2020 Feb 27;13:111. doi: 10.1186/s13104-020-04962-1 (PMC7045480; doi:10.1186/s13104-020-04962-1)
Supplement: Supplementary file 2 — Additional file 2. Anonymous evaluation survey accident prevention in children and pediatric cardiopulmonary resuscitation course. [file 13104_2020_4962_MOESM2_ESM.docx]

**Additional file 2**

**Anonymous evaluation survey accident prevention in children and pediatric cardiopulmonary resuscitation course**

1. THEORETICAL CONTENTS
2. The theoretical contents of the lectures have been:
3. Very poor
4. Poor
5. Fair
6. Good
7. Excellent
8. The amount of theoretical background information has been:
9. Adequate
10. Scant
11. Excessive
12. The exposition of the theoretical contents of the matter during the lectures has been:
13. Very poor
14. Poor
15. Fair
16. Good
17. Excellent
18. Please rate from 1 (very poor) to 10 (excellent) each of the LECTURES
19. Cardiopulmonary resuscitation: Concept and Epidemiology
20. Basic cardiopulmonary resuscitation
21. If any of the lectures contents or the way of presenting them have seemed inadequate, please write the reasons
22. Do you propose any modification in the theoretical content of the classes for future courses?
23. PRACTICAL CONTENTS
24. The hands-on workshops with the mannequins have been:
25. Very poor
26. Poor
27. Fair
28. Good
29. Excellent
30. The time to practice of each student has been:
31. Adequate
32. Scant
33. Excessive
34. The number of students in each workshop has been:
35. Adequate
36. Scant
37. Excessive
38. Please rate from 1 (very poor) to 10 (excellent) each of the WORKSHOPS
39. Accidents prevention and First Aid Kit
40. First aid in the event of accidents
41. Basic pediatric cardiopulmonary resuscitation
42. If any of the workshops have seemed inadequate, please write the reasons
43. Do you propose any modification in the workshops for future courses?
44. FACULTY
45. The teaching competence of the faculty has been:
46. Very poor
47. Poor
48. Fair
49. Good
50. Excellent
51. The consistency between theoretical and practical knowledge, and among the faculty has been:
52. Very poor
53. Poor
54. Fair
55. Good
56. Excellent
57. TIME SCHEDULE AND COURSE DURATION
58. The duration of the course has been:
59. Adequate
60. Too short
61. Too long
62. The time schedule of the course has been:
63. Adequate
64. Excessive hours per day
65. Few hours per day
66. STUDENT SELF EVALUATION
67. Please enter your profession.
68. Have you previously completed a CPR course?

YES NO

What kind of course?

1. Have you previously performed any basic cardiopulmonary resuscitation? If so, please describe the number of times and circumstances (kind of patient, setting, number of rescuers)

YES NO

1. Do you consider yourself prepared to provide cardiopulmonary resuscitation to a child?

YES NO

Why?

1. Do you think that participating in refresher courses periodically would be convenient?

YES NO

1. How often?
2. Never
3. 3 months
4. 6 months
5. 1 year
6. 2 years
7. 5 years
8. 10 years
9. Would you be interested in been trained as an instructor in pediatric CPR and accident prevention courses?

YES NO
